# Invisible experts: a systematic review & thematic synthesis of informal carer experiences of inpatient mental health care

**DOI:** 10.1186/s12888-022-03872-9

**Published:** 2022-05-20

**Authors:** Nada Abou Seif, Lisa Wood, Nicola Morant

**Affiliations:** Division of Psychiatry, University College London, 149 Tottenham Court Road, London, W1T 7NF UK

## Abstract

**Background:**

The negative impact of caregiving on carers’ physical and psychological wellbeing is well documented. Carers of mental health inpatients have particularly negative experiences and largely report being dissatisfied with how they and their loved one are treated during inpatient care. It remains unclear why, despite policies intended to improve inpatient experiences. A comprehensive review of carers’ inpatient experiences is needed to understand carer needs. As such, we aimed to conduct a systematic review and thematic synthesis of carer experiences of inpatient mental health care.

**Methods:**

We searched MEDLINE, PsycINFO, Embase and CINAHL for qualitative studies examining carer experiences of mental health inpatient care. Searches were supplemented by reference list screening and forward citation tracking of included studies. Results were synthesised using thematic synthesis. Our protocol was registered on PROSPERO (CRD42020197904) and our review followed Preferred Reporting Items for Systematic Reviews and Meta-Analyses (PRISMA) guidelines.

**Findings:**

Twelve studies were included from 6 countries. Four themes were identified: the emotional journey of inpatient care; invisible experts; carer concerns about quality of care for their loved one; and relationships and partnership between carers, service users and staff.

**Interpretation:**

Greater attention should be paid to ensure carers are well-supported, well-informed, and included in care. More emphasis must be placed on fostering positive relationships between carers, service users and staff and in facilitating continuity of care across inpatient and community services to provide carers with a sense of security and predictability. Further research is needed to explore differences in experiences based on carer and service user characteristics and global context, alongside co-production with carers to develop and evaluate future guidelines and policies.

**Supplementary Information:**

The online version contains supplementary material available at 10.1186/s12888-022-03872-9.

## Research in context

### Evidence before this study

Caregivers of mental health inpatients consistently report negative experiences with inpatient services. It remains unclear why this persists, despite policies intended to improve their experiences. Existing reviews have not considered carer views of the experience itself or excluded experiences with voluntary hospitalisations. As such, this review aimed to explore carer experiences of routine inpatient mental healthcare. MEDLINE, PsycINFO, Embase and the Cumulative Index to Nursing and Allied Health Literature (CINAHL) were searched from inception to February 2021. Studies were included if they employed qualitative methodology, had a sample consisting of at least 90% carers, reported on the experiences of carers with adult (18 +) mental health inpatient care and were published in English in a peer-reviewed journal. All studies were of high quality.

### Added value of this study

This systematic review and thematic synthesis suggest that carer experiences with inpatient services continue to be characterized by emotional turmoil, a lack of support and exclusion from and dissatisfaction with care. Furthermore, it was suggested that positive relationships between staff and carers and between staff and service users, continuity of care, and acknowledging carers as both humans and experts are essential to improving carer experiences with inpatient care.

### Implications of all the available evidence

Our review suggests that there is a need for greater continuity across inpatient and community services and that inpatient staff should make greater attempts to foster positive relationships and to ensure carers are well supported, informed and included. Further research is needed to explore the impact of service user and carer characteristics on carer experiences. There is also a need for co-production with carers in the development and evaluation of policies intended to improve inpatient services to meet carer needs.

## Introduction

The shift in psychiatric care towards community care and deinstitutionalisation in many countries [1–4] has resulted in family members caring for loved ones with mental health difficulties [5–7]. In the UK alone, there are an estimated 6.8 million informal carers who, through their unpaid care, save the UK approximately £132 billion annually [8]. There is clear evidence of the benefits of carer involvement for individuals experiencing mental health difficulties, with higher carer involvement being associated with reductions in symptoms, risk of relapse and inpatient admissions [9–12]. However, caregiving can have pervasive and enduring detrimental effects with higher rates of common mental disorder and physical health difficulties in carers [13–15].

Deinstitutionalisation has also led to significant changes in inpatient mental health services, which provide support to those experiencing mental health crises so severe they cannot be managed or treated in the community [16, 17]. In the UK, these units have seen a 73% reduction in beds from 1987 to 2019 [18]. In turn, this has led to a significant change in the profile of mental health inpatients, with only the most severe presentations, often with comorbidities and social difficulties, being admitted [18, 19]. Similar shifts in mental healthcare have been seen internationally, such as with the Brazilian Psychiatric Reform [20]. Thus, carers of inpatients are likely to be experiencing additional challenges associated with the severity of their loved one’s illness. Instead of inpatient admission being a time when carers obtain respite from caregiving, research suggests that this may be a time of increased stress [20]. Research has demonstrated that this may be linked to a lack of information being provided to carers, likely due to patient confidentiality and the time-limited nature of inpatient stays preventing staff from providing carers with more information [21, 22]. Moreover, it is a time where their loved one may be experiencing acute emotional distress, high risk of harm to themselves or others, and in some cases relational conflict. There is also extensive evidence that service users are generally dissatisfied with inpatient care due to restrictive practices, limited access to psychosocial interventions, and a lack of collaborative care, beliefs which are likely to be endorsed by their carers [10]. Furthermore, inpatient carers report significantly higher burden than carers of outpatients, with carer burden being significantly associated with perceived unmet needs of the service user [23].

In recognition of these issues, policies such as the National Carers Strategy and NHS Triangle of Care have encouraged the support of and collaboration with carers [24, 25]. However, research conducted internationally has demonstrated that carers’ qualitative accounts continue to depict predominantly negative experiences of inpatient services, characterized by a lack of support and exclusion from their loved one’s care [22, 26–33]. For example, carers report feeling excluded from the processes of admission, treatment and discharge planning [26, 30]. It is likely that these negative experiences play a role in the increased stress and burden experienced by inpatient carers. Nonetheless, carers in many countries consistently indicate a desire to be valued, to work in collaboration with inpatient staff, and for greater support in managing financial burdens, their physical and mental health, and their loved one’s mental health [27, 33–37].

There have been a number of qualitative reviews which have examined the impact of psychiatric hospitalisation on carers including mixed methodology papers [38], and qualitative-focused reviews examining carer experiences of mental health crises [39] and detention under mental health legislation [40]. However, these reviews did not examine the subjective experience of family and carers using qualitative synthesis methodology [38], or excluded experiences with voluntary hospitalisations [39, 40]. However, as routine inpatient care includes both voluntary and involuntary admissions [41], to exclude carer experiences of either is to paint an incomplete picture. This review seeks to fill this gap and aims to explore carer experiences of routine inpatient mental healthcare.

## Method

### Design

We conducted a systematic review and thematic synthesis of qualitative literature exploring carer experiences of mental health inpatient care. Our protocol was registered on PROSPERO (CRD42020197904) and the review was conducted in accordance with best practice guidance as outlined by the Preferred Reporting Items for Systematic Reviews and Meta-Analyses (PRISMA) [42].

### Search strategy and selection criteria

Searches were conducted on Embase, Medline, PsycINFO (all via Ovid) and CINAHL (via EBSCOhost) in June 2020, and updated in February 2021. This allowed us to cover a broad range of multidisciplinary clinical evidence. Searches contained keywords pertaining to the relevant sample (carers, caregivers, parents, fathers, mothers, spouses, wives, husbands), mental health context (inpatient, acute, psychiatric, mental health, hospitalisation, ward), and study design (qualitative, interviews, focus groups). Full search strategies for each database are available in the supplementary material. Searches were supplemented by screening reference lists and forward citation tracking of included studies to reduce the chance of missing relevant studies.

Studies were included if they: (a) used semi-structured interviews or focus groups; (b) had a sample that consisted of majority carers (at least 90%); (c) reported on the experiences of carers with adult (18 +) mental health inpatient care (d) were published in English in a peer-reviewed journal.

Studies were excluded if they: (a) examined a discrete component of inpatient care e.g., admission under the Mental Health Act (b) reported on experiences with services for children and young people, intellectual and/or learning disabilities, older adults or forensic services; (c) utilised surveys or questionnaires; (d) were reported in conference abstracts, books, editorials or general commentary.

### Data screening and extraction

All titles and abstracts were screened by NAS (using EndNote), with a random 20% being reviewed by an additional independent reviewer. Full texts were then screened for inclusion by NAS with any disagreements discussed with LW. Where additional information was required to determine eligibility, authors were contacted for clarification. The following information was extracted from each included study: (1) authors, (2) country where research was conducted, (3) study aim, (4) sample size & characteristics (including age, nature of relationship with service user and gender), (5) the data collection method and (6) the analytic approach utilised.

### Quality appraisal

Quality appraisal was conducted using the Critical Appraisal Skills Programme (CASP) qualitative checklist, which is a deviation from the registered protocol [43]. We chose to use the CASP qualitative checklist as it is the most commonly used tool for quality appraisal in health and social care related qualitative research [44–46] and has been endorsed by both the Cochrane Qualitative and Implementation Methods Group and the World Health Organisation [44, 46–48]. This checklist examines whether studies included sufficient description and justification of the chosen methods of data collection, sampling, and analytical approach, as well as whether sufficient attention was given to ethics and the role of the researchers involved. In accordance with guidelines for thematic synthesis, no studies were excluded on the basis of quality [48, 49].

### Data synthesis

The thematic synthesis of qualitative research in psychiatry as outlined by Lachal et al. [48] and adapted from Thomas & Harden [49] was used to guide analysis. The results section of each study, including verbatim carer quotes and author analysis, was extracted and used as data. This was then exported into NVivo12 for thematic analysis [50]. A critical realist approach was taken and all data (author analysis and carer quotes) within the included studies were analysed from an inductive data-driven position. To start, data was read and re-read by NAS to achieve sufficient familiarisation, and ultimately immersion, with the data. An initial coding frame was developed using half of the included studies and was further developed through the identification of shared themes while coding the second half of studies. Line by line coding was undertaken and codes were highlighted if they were thought to represent carers experiences of inpatient care. A code was usually represented by a short phrase e.g. "feelings of guilt". Codes were then collapsed and grouped together to construct descriptive themes. The descriptive themes were then synthesised across studies to develop overarching analytical themes and sub-themes that would capture carer experiences of inpatient mental healthcare.

This review was primarily conducted by NAS, who was a research student with no prior experience of inpatient care. She was supervised by LW, a clinical psychologist and researcher who works in acute mental health inpatient settings with carers and NM, an academic and qualitative methodologist who undertakes mental health research. The lead researcher kept reflexive notes to identify and address any biases brought to the analysis by the research team.

## Results

### Search results

**Fig. 1:**
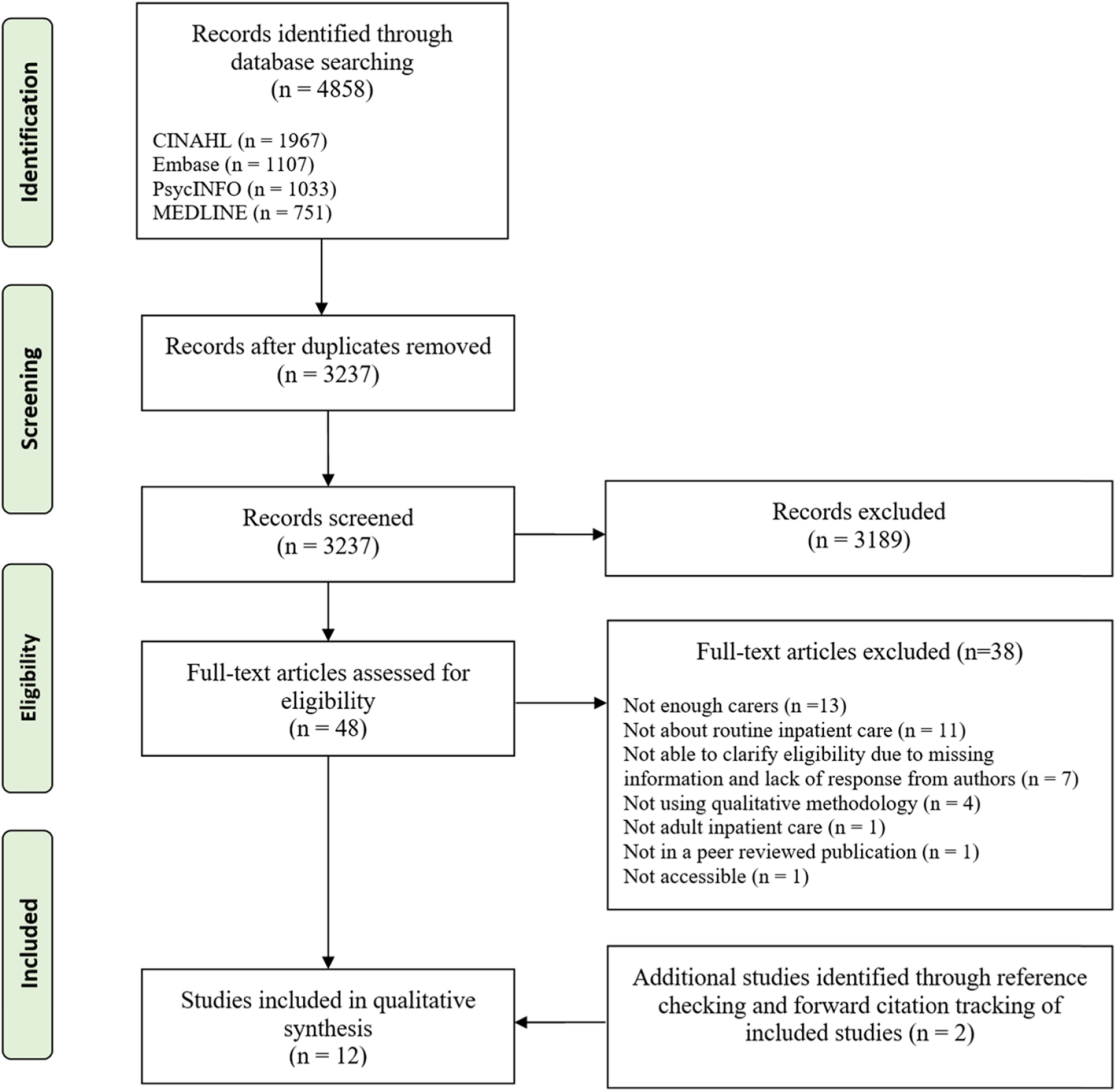
PRISMA Diagram

The search yielded 3237 articles after the removal of duplicates. Out of the 48 studies selected for full-text screening, a further 38 were excluded due to not meeting the eligibility criteria. Two additional eligible studies were identified using forward citation tracking, resulting in a total of 12 studies included in the final synthesis. The search process is outlined in Fig. 1.

### Study characteristics

Table 1 shows the characteristics of included studies. The total number of carers included was *n* = 165, with sample sizes ranging from *n* = 3 to *n* = 31. Carers were predominantly female (60%), parents (63%), and from White ethnic backgrounds (68%). For data collection, studies utilised interviews (*n* = 10), discussion groups (*n* = 1) and a combination of both (*n* = 1). All included studies were of high quality, with CASP scores ranging from 8 to 10 (median score = 8) out of a maximum score of 10.

**Table 1:** Study characteristics

Authors	Country	Aim	Sample size & characteristics	Data collection method	Analytic approach	CASP Score (max 10)
Clarke & Winsor (2010) [35]	Canada	Exploring the perceptions and needs of parents during a young person’s first psychiatric hospitalization	*N* = 10 Nature of relationship: 9 mothers,1 father M/F: 9/1 Age 40–59	Semi-structured interviews	Morse & Field’s four processes	8
Crisanti (2000) [28]	Canada	Examining mothers’ experiences with the involuntary hospitalization of their adult child with schizophrenia	*N* = 3 Nature of relationship: 3 mothers M/F: 0/3 Patient diagnosis: 3 schizophrenia Patient illness duration: 12–20 years	Semi-structured interviews	Phenomenology (VanKaam’s)	8
Da Silva Andrade et al. (2016) [20]	Brazil	Examining the feelings of relatives of individuals admitted to a psychiatric emergency care unit	*N* = 20 M/F: 9/11 Age: 40–65	Semi-structured interviews	Thematic content analysis (Bardin’s)	8
Guimaraes et al. (2019) [51]	Brazil	Exploring the expectations of family members of alcoholics admitted to the Psychiatric Hospitalization Unit	*N* = 15 Nature of relationship: 4 mothers, 4 brothers, 2 sisters, 2 sons, 1 daughter, 1 grandfather, 1 son-in-law M/F: 8/7 Age: 25–73 –- Patient diagnosis: 15 alcohol use disorder	Semi-structured interviews	Phenomenological Sociology	8
Hickman et al. (2016) [52]	UK	Examining the experiential impact of hospitalisation on the parents of young people with early psychosis	*N* = 6 Nature of relationship: 4 mothers and 2 fathers M/F: 4/2 –- Patient diagnosis: 6 psychosis	Semi-structured interviews	Interpretative Phenomenological Analysis	9
Jagannathan et al. (2011) [34]	India	Exploring the needs of caregivers of inpatients with schizophrenia	*N* = 30 Nature of relationship: 21 parents, 4 siblings, 3 “other", 2 spouses M/F: 13/17 –- Patient diagnosis: 30 schizophrenia	Focus group discussions	“Iteration”	8
Jankovic et al. (2011) [26]	UK	Examining family caregivers’ experience of the involuntary admission of their relative	*N* = 31 Nature of relationship: 16 parents, 7 partners, 4 siblings, 2 children, 1 grandmother, 1 “elderly relative” M/F: 12/19 Age: 18–59 Ethnicity: 21 White, 10 Asian, Black, or Mixed –- Patient diagnosis (on discharge): 8 schizophrenia, 6 bipolar disorder, 2 recurrent depressive disorder, 1 schizoaffective disorder, 1 “manic episode”, 1 borderline personality disorder, 1 “no mental illness on discharge”, 2 “unavailable” First hospitalisation: 12	Semi-structured interviews	Thematic analysis	9
Fernandes Moll et al. (2018) [53]	Brazil	Investigating the perceptions and expectations of family members/caregivers of psychiatric nursing care	*N* = 10 Nature of relationship: 50% parents M/F: 7/3 Average age: 58.8 –- Patient diagnosis: 6 schizophrenia, 3 depression, 1 drug abuse	Semi-structured interviews	Content analysis	8
Park & Lee (2017) [54]	South Korea	Exploring Korean sibling caregivers’ experiences with siblings with schizophrenia that had been hospitalised in an inpatient psychiatric unit	*N* = 8 Nature of relationship: 8 siblings M/F: 3/5 Age: 20 s to 40 s –- Patient diagnosis: 3 schizophrenia	Semi-structured interviews, supplemented by field notes and memos	Descriptive Phenomenology (Colaizzi’s)	8
Wilkinson & McAndrew (2008) [27]	UK	Examining carers’ perceptions of their level of involvement in acute inpatient care	*N* = 4 Nature of relationship: 2 mothers, 1 wife, 1 husband M/F: 1/3 –- Patient diagnosis: 2 paranoid schizophrenia, 1 depression, 1 bipolar disorder	In-depth interview	Hermeneutic Phenomenology (Heidegger’s)	10
Wood et al. (2013) [55]	UK	Examining the extent to which carers are positioned as ‘outsiders’ in inpatient settings, and how ‘permeable’ hospitals are	*N* = 9 carers (and 1 staff)	Discussion groups and semi-structured interviews	Thematic analysis	9
Wyder et al. (2018) [56]	Australia	Exploring the experiences of families of involuntary mental health admissions	*N* = 19 Nature of relationship: 9 mothers, 6 fathers, 3 partners, 1 sibling M/F: 7/12 –- Patient diagnosis: 6 schizophrenia, 4 psychotic illness, 4 drug-induced psychosis, 1 bipolar,1 organic brain disease	Semi-structured interviews, with 1 participant emailing in information	“General inductive approach”	8

### Thematic synthesis

Four overarching themes were identified: the emotional journey of inpatient care, invisible experts, carer views on quality of care for their loved one, and relationships and partnership between carers, service users and staff (Table 2).

**Table 2:** Summary of themes

Themes	References
The emotional journey of inpatient care	[20, 26–28, 34, 35, 51, 52, 54, 56]
Invisible experts	[26–28, 35, 52, 55, 56]
Carer concerns about quality of care for their loved one	[20, 26, 28, 52, 53, 55, 56]
Relationships and partnership between carers, service users and staff	[20, 26, 28, 35, 51–53, 55, 56]

## The emotional journey of inpatient care

The emotional journey of inpatient care was discussed in nearly all studies (*k* = 10) [20, 26–28, 34, 35, 51, 52, 54, 56].

### The build up to hospitalisation

The build up to hospitalisation was described as distressing and overwhelming, as carers find themselves having to juggle managing the deterioration of their loved one’s condition, increased levels of risk of harm, while navigating the mental health system to get help. Carers report feeling powerlessness and frustration that help was seemingly only made available once their loved one had deteriorated to a point where hospitalisation was inevitable.

“I mean one day he had me in tears, I had to walk out of the house and I just walked into the police station and I spoke to somebody on the desk, and they gave me a little bit of advice and they told me who to contact and stuff, and the next day I rang, I actually spoke to somebody but even that was a long process. I phoned them one day and they said they would get back to me and I said like, I need help now not like tomorrow or next week. I think like they got back to me three months later, it was really, really hard to get any kind of help to start with.” (Carer) [26]

### Hospital care

Once their loved one was hospitalised, carers reported experiencing a mixture of conflicting emotions such as relief, guilt, fear, and hope. This was due to carers being hopeful to obtain some respite from caregiving, and for their loved one to receive appropriate treatment and containment, but also guilt and fear about the safety and quality of care their loved one would receive.

“Participants’ accounts of hospitalisation framed it overwhelmingly as an appropriate intervention that brought relief and respite. The young person was understood to be physically contained, with access to appropriate treatment, and hospital was seen as a place of safety, for self and society.” (Authors) [52]

“The mothers expressed that they often felt as if they were being judged as parents who were trying to ‘get rid of the problem.’” (Author & Carers) [28]

## Invisible experts

Carers’ feelings of invisibility and neglect by mental health professionals, as well as their exclusion from their loved one’s care during hospitalisation were reported as central to carers’ experiences with inpatient care across studies that took place in the UK, Canada and Australia (*k* = 7) [26–28, 35, 52, 55, 56].

### The invisibility & neglect of carer needs

Despite the significant emotional toll of the hospitalisation and a heightened need for support, carers report that their needs were seldom acknowledged and strongly perceived inpatient staff as unsupportive. They were not offered support or asked about their own needs by staff. Moreover, carers felt uninformed about their loved one’s treatment and wanted more information on mental illness and how to manage illness-related behaviours.

*“Interestingly, the families clearly did not perceive staff as being supportive…they were seldom, if ever, acknowledged when they visited their child…when asked outright if they found the health care providers to be supportive, they answered resoundingly, ‘No’.” (Authors) *[35]

Although rare (*k* = 3), when carers did feel supported and were given the opportunity to obtain clear and accessible information, they found the inpatient experience more positive and reported tremendous relief [27, 53, 55].

### Exclusion of carer expertise from loved one’s care

Despite being expected to care for the service user throughout hospitalisation, and being experts in their own right, carers felt excluded from inpatient care. Carers reported being informed of decisions regarding their loved one’s care last minute or after the fact, if at all, whether by phone or in person. Due to their desire to be included in the decision-making process, carers found this lack of transparent communication and information particularly frustrating. While carers did not frequently report being invited to care planning meetings, even when they were invited, they describe feeling a resignation to their inability to affect change as their views were largely dismissed by staff. Carers felt that their expertise was not drawn upon or included in clinical decision making. This was particularly contentious during discharge planning.

*“I wasn’t involved, I was an afterthought . . . no one told us anything, no one rang to keep us up to date with the plan of care. I only found out that he (son) had been started on an injection when he rang to tell me that he’d had a needle in his bum . . . How can I look after him at home if I don’t know what I’m supposed to be doing?” (Carer) *[27]

Another source of contention was the seemingly non-reciprocal nature of information sharing, wherein carers were being relied on to provide information, but not being provided any in return. Confidentiality was cited as an issue here. While carers acknowledged that this may serve to protect the service user’s privacy, they felt that, for both their wellbeing and the service users, it was their right to be informed.

*“Before they will talk to me about anything, they always say is it alright if I talk to your mother which is fine because it’s patient confidentiality. But you know, when I’m the one that’s at risk, I expect a bit of a say so in it. That’s fine if you’ve got him a safe place and he’s being looked after, but when he’s out in the community with me, then I expect a say in what goes on.” (Carer) *[26]

Carers who were included in the treatment process (*k* = 2) had considerably more positive views of the inpatient experience and felt empowered and confident to care for their loved one after discharge [27, 56].

## Carer concerns about quality of care for their loved one

Carers reported concerns about the quality of inpatient care across the majority of studies (*k* = 7) [20, 26, 28, 52, 53, 55, 56]. Carers spoke of dissatisfaction relating to delays in admission, unmet service user needs, staff competence, the duration of hospitalisation, safety and the lack of space. As a result, carers felt that appropriate and timely help was not being provided. Carers were also displeased with the heavy focus on medicating the service user, noting that no one really spoke to their loved one.

“All she does is see a doctor once or twice a week. There’s no counsellor brought in […] She seriously needs to talk to somebody, not for 10 minutes, how you’re going, how you’re feeling, are you still seeing anything? That’s all she gets. She’s never actually sat down with anybody and just talked about anything.” (Carer) [56]

Moreover, carers’ views on staff competence and the degree to which they trusted the treatment plan played an important role in influencing views on care. They consistently spoke of disagreement with the duration of hospitalisation, and that sufficient help was not actually received during hospitalisation or at the point od discharge. Carers wanted continuity of care and support for themselves and their loved one throughout the care journey.

“This disagreement with the clinicians’ assessment regarding the level of professional support required was noticeable before discharge and it contributed to the burden of care shifted from services…More specifically, family caregivers commonly believed that the patient should have been admitted earlier or discharged later, and this was a concern reported mainly by family caregivers of patients with previous hospital admissions.” (Authors) [26]

## Relationships and partnership between carers, service users and staff

Carers describe relationships between carers, service users and staff as being integral to their experiences of inpatient care (*k* = 9) [20, 26, 28, 35, 51–53, 55, 56].

### Carers & their loved one: Distance and strain

Carers reported that with the hospitalisation, a strain was placed on their relationship with the service user. For some, this was linked to blame, either from the service user blaming the carer for the hospitalisation, or the carer blaming the service user for their mental health difficulties.

*“He had this great hatred of me, whatever it was, so it was very difficult for me, it was a great hate. And I think it stemmed from I was the one, I actually put him into the hospital.” (Carer) *[52]

Others cited an inability to visit their loved one as often as desired due to practical difficulties such as other life responsibilities or rigid visiting hours, which was distressing for both parties. Flexibility and support from inpatient staff were valued by carers in relation to these practical barriers.

*“The more complicated public transport route, using more buses, the extra cost of the travelling, and the fact that “sometimes your benefits get reduced after you have been in [the hospital] for so long” were all issues relating to the location of the hospital, which was felt to prohibit regular visiting...” (Authors & Carer) *[55]

### Carers & staff: a desire for partnership

Carers’ relationship with staff also played an important role in their experience. They described the relationship as marked with tension and reported sensing a divide of “us vs them”, wherein they were viewed as threats, challenging, or nuisances. Nonetheless, carers consistently expressed a desire for partnership with staff, citing their belief that this would ultimately foster a better inpatient experience for both carers and service users. Carers felt that continuity of staff would facilitate partnership, as they would feel more at ease expressing themselves with staff they were familiar with.

*“It’s about working together, the team knowing that I have valuable things to contribute and *vice versa*, because we all want the same at the end of the day.” (Carer) *[27]

### Service users & staff: a need for affection

Equally important to carers was the relationship between service users and staff. They spoke of wanting staff to be more affectionate and caring to their loved one, to actively listen and talk to them, and to help them with self-care. When staff were compassionate and offered a caring human response, it helped patients feel more comfortable and engaged in care which was reassuring for carers. However, some carers alluded to the lack of time as being a barrier to staff being able to do this.

*Amy was distressed that her husband would sometimes get “very upset going back to the [Old] hospital” after she had accompanied him outside the hospital on “leave”…if a member of staff took the time to talk to her husband, to welcome him back … her “husband would go in bouncy instead of going up to his room and crying, and that made a huge difference”. (Authors & Carer)* [55]

^*“*^*"All she does is see a doctor once or twice a week. There’s no counsellor brought in […] She seriously needs to talk to somebody” (Carer) *[21]

## Discussion

### Summary of findings

Our thematic synthesis highlighted the distressing and overwhelming nature of the buildup to hospitalisation for carers. They describe struggling to manage the deterioration of their loved one’s condition while attempting to obtain help within a confusing mental health system. Once their loved one was hospitalised, carers describe emotional conflict, with initial relief associated with respite from caregiving but also guilt and fear. Carers were also quickly disillusioned with the quality of care provided, particularly the lack of timely and appropriate help. These findings have been identified in similar reviews [38–40].

Our synthesis suggests that carers’ lack of support and exclusion from their loved one’s care are integral to their experience. Carers report perceiving inpatient staff as insensitive to their emotional needs and felt unsupported, uninformed, and unacknowledged throughout the hospitalisation. Carers desperately wanted more information on the illness and treatment plan and wanted greater involvement throughout treatment and discharge planning. This lack of participation and information is also consistent with the aforementioned reviews [39, 40].

### Clinical implications

Our findings suggest that collaborative relationships may hold the potential to transform inpatient experiences. Carers describe a distance and strain placed on their relationship with the service user, and the distress this causes for both parties, supporting previous reviews [38, 40]. Similarly, carers often described their relationship with staff as marked by tension and dismissal. Although rare, when carers felt included, they describe a much more positive inpatient experience, as well as confidence in their own caregiving abilities, in line with previous research [40]. An important finding derived from our synthesis is the importance placed by carers on the relationship between service users and staff, with carers wanting staff to be more caring to their loved one. When this occurred, they described it as therapeutic both to them and the service user.

The importance of acknowledging carers, both as humans and experts, was suggested as essential to improving carer experiences with inpatient care. As such, greater attempts should be made by professionals to understand the unique needs of carers and how to best meet them, particularly soon after the hospitalisation of their loved one, as this was suggested to be a particularly vulnerable time. There is a need for more accessible information; carers should be provided with psychoeducation regarding their loved one, as well as information on how to manage their own wellbeing. Additionally, inpatient staff should be more proactive in attempts to include carers in care planning, particularly surrounding discharge. Within this, professionals should acknowledge carers’ expertise on their loved one, which would in turn foster a sense of partnership and help break down the divide reported by carers between them and staff. There is also a need for greater continuity across inpatient and community services. While continuity of care is often thought of in relation to its importance to service users [57], our findings suggest that it is equally important to carers.

#### Strengths, limitations and future research

Future research should attempt to explore whether differences in carer and service user characteristics influence carer experiences. An area of focus should be the examination of the experiences of carers of ethnic minorities, as this group tends to be underrepresented. Additionally, as many participants in studies in this area tend to be parents, and particularly mothers, greater attempts should be made to explore different carer-service user relationships. Future research would also benefit from greater co-production with carers in the development and evaluation of policies that intend to help inpatient services better meet carer needs.

The primary strength of this study was that it followed best-practice guidelines in undertaking systematic reviews. For example, this review was registered with PROSPERO, followed PRISMA guidelines, and searches were conducted across an array of databases, allowing us to cover a broad range of clinical evidence. Moreover, all the included studies included in the review were of high quality, as demonstrated by high scores on the CASP checklist.

However, there are several limitations to be noted. First, as thematic synthesis is a form of secondary analysis, our analysis is dependent upon study authors’ interpretation and presentation of their original qualitative data, which may lead to bias. This also meant that we were unable to analyse differences in experiences based on the characteristics of carers or service users (e.g., ethnicity, diagnosis), as these distinctions were not made in the included studies. Second, the lack of detail within included studies surrounding certain issues raised by carers limited further interpretation. For example, in “4C: Service users & staff: A need for affection”, the limited data made it difficult to infer why carers thought there was a lack of affection, making it difficult to suggest remedial strategies. Further research should explore the reasons for this. Third, despite the consistency of themes across studies, their limited geographical spread makes it difficult to determine whether our findings would be representative of carer experiences in different geographical contexts. Similarly, we were unable to make comparisons of the diverse models of mental healthcare due to the limited information provided within the studies. Further research should seek to examine how different mental healthcare models influence carer experiences. Fourth, as we only included studies published in peer-reviewed publications it is possible that we have missed relevant findings. There is evidence suggesting particular difficulty in recruiting carers to research studies [58], and as such, it is possible that our findings omit important perspectives in the grey literature that may have been explored within research conducted in non-academic settings such as charities. Finally, we were unable to obtain input from individuals with lived experience of informal caregiving in the development of the project due to limited time and resources.

In summary, our review highlights that carer experiences are marked by emotional turmoil, a lack of support and exclusion from and dissatisfaction with care. Our review suggests that there is a need for greater continuity across inpatient and community services and that inpatient staff must make greater attempts to foster positive relationships and to ensure carers are well supported, informed and included.

## Supplementary Information

**Additional file 1: Supplementary Material.** Search Strategies

## Data Availability

Data availability is not applicable to this article as no new data were created or analysed in this study.
