# Supplementary material for: Invisible experts: a systematic review & thematic synthesis of informal carer experiences of inpatient mental health care
Source: BMC Psychiatry. 2022 May 20;22:347. doi: 10.1186/s12888-022-03872-9 (PMC9121622; doi:10.1186/s12888-022-03872-9)
Supplement: Supplementary file 1 — Additional file 1: Supplementary Material. Search Strategies [file 12888_2022_3872_MOESM1_ESM.docx]

**Supplementary Material: Search Strategies**

| **MEDLINE (Ovid)** | | |
| --- | --- | --- |
|  | **Search term** | **Results** |
| 1 | Caregivers/ | 36315 |
| 2 | Family/ | 75615 |
| 3 | Parents/ | 63318 |
| 4 | Spouses/ | 10069 |
| 5 | (Famil* or carer* or caregiver* or relative* or parent* or mother* or father* or spouse* or wife or wives or husband*).tw. | 2889270 |
| 6 | 1 or 2 or 3 or 4 or 5 | 2927189 |
| 7 | Inpatients/ | 21749 |
| 8 | Hospitalization/ | 106634 |
| 9 | 7 or 8 | 125679 |
| 10 | exp Psychiatry/ | 103823 |
| 11 | 9 and 10 | 2342 |
| 12 | Hospitals, Psychiatric/ or Involuntary Treatment, Psychiatric/ | 2522 |
| 13 | ((Psychiatr* or mental) adj4 (Inpatient* or in-patient* or hospital* or acute or ward*)).tw. | 41048 |
| 14 | 11 or 12 or 13 | 57395 |
| 15 | ((("semi-structured" or semistructured or unstructured or informal or "in-depth" or indepth or "face-to-face" or structured or guide) adj2 (interview* or discussion* or questionnaire*)) or (focus group* or qualitative or ethnograph* or fieldwork or "field work" or "key informant")).tw,kw. or interviews as topic/ or focus groups/ or narration/ or qualitative research | 386470 |
| 16 | 6 and 14 and 15 | 751 |

| **PsycINFO (Ovid)** | | |
| --- | --- | --- |
|  | **Search term** | **Results** |
| 1 | Caregivers/ | 28489 |
| 2 | Family/ | 48663 |
| 3 | Parents/ | 39778 |
| 4 | Spouses/ | 11757 |
| 5 | (Famil* or carer* or caregiver* or relative* or parent* or mother* or father* or spouse* or wife or wives or husband*).tw. | 959407 |
| 6 | 1 or 2 or 3 or 4 or 5 | 963449 |
| 7 | exp Psychiatric Patients/ or exp Hospitalized Patients/ or exp Psychiatric Hospitalization/ | 46641 |
| 8 | ((Psychiatr* or mental) adj4 (Inpatient* or in-patient* or hospital* or acute or ward*)).tw. | 46201 |
| 9 | 7 or 8 | 75551 |
| 10 | exp Psychiatry/ | 103823 |
| 11 | 9 and 10 | 2342 |
| 12 | Hospitals, Psychiatric/ or Involuntary Treatment, Psychiatric/ | 2522 |
| 13 | ((("semi-structured" or semistructured or unstructured or informal or "in-depth" or indepth or "face-to-face" or structured or guide) adj2 (interview* or discussion* or questionnaire*)) or (focus group* or qualitative or ethnograph* or fieldwork or "field work" or "key informant")).tw. or interviews as topic/ or focus groups/ or narration/ or qualitative research | 275789 |
| 14 | 6 and 9 and 10 (1033) | 1033 |

| **CINAHL (EBSCOhost)** | | |
| --- | --- | --- |
|  | **Search term** | **Results** |
| 1 | MH "Caregivers" | 37,838 |
| 2 | MH "Family" | 45,042 |
| 3 | MH “Parents” | 46,979 |
| 4 | MH” Spouses” | 11,594 |
| 5 | Famil* or carer* or caregiver* or relative* or parent* or mother* or father* or spouse* or wife or wives or husband* | 878,818 |
| 6 | 1 or 2 or 3 or 4 or 5 | 878,818 |
| 7 | (MH "Hospitals, Psychiatric") OR (MH "Psychiatric Units") OR (MH "Psychiatric Patients") | 21,246 |
| 8 | ((Psychiatr* or mental) N4 (Inpatient* or in-patient* or hospital* or acute or ward*)) | 47,039 |
| 9 | 7 or 8 | 48,002 |
| 10 | ((("semi-structured" or semistructured or unstructured or informal or "in-depth" or indepth or "face-to-face" or structured or guide) N2 (interview* or discussion* or questionnaire*)) or (focus group* or qualitative or ethnograph* or fieldwork or "field work" or "key informant")) or (“qualitative research”) | 315,476 |
| 11 | 6 and 9 and 10 | 1,967 |

| **Embase** | | |
| --- | --- | --- |
|  | **Search term** | **Results** |
| 1 | Caregivers/ | 58024 |
| 2 | Family/ | 87206 |
| 3 | Parents/ | 73610 |
| 4 | Spouses/ | 15131 |
| 5 | (Famil* or carer* or caregiver* or relative* or parent* or mother* or father* or spouse* or wife or wives or husband*).tw. | 3465618 |
| 6 | 1 or 2 or 3 or 4 or 5 | 3502196 |
| 7 | Inpatients/ | 139713 |
| 8 | Hospitalization/ | 364992 |
| 9 | 7 or 8 | 481773 |
| 10 | exp Psychiatry/ | 119656 |
| 11 | 9 and 10 | 6085 |
| 12 | Hospitals, Psychiatric/ or Involuntary Treatment, Psychiatric/ | 20364 |
| 13 | ((Psychiatr* or mental) adj4 (Inpatient* or in-patient* or hospital* or acute or ward*)).tw. | 50148 |
| 14 | 11 or 12 or 13 | 63825 |
| 15 | ((("semi-structured" or semistructured or unstructured or informal or "in-depth" or indepth or "face-to-face" or structured or guide) adj2 (interview* or discussion* or questionnaire*)) or (focus group* or qualitative or ethnograph* or fieldwork or "field work" or "key informant")).tw,kw. or interviews as topic/ or focus groups/ or narration/ or qualitative research | 434923 |
| 16 | 6 and 14 and 15 | 1107 |
